# Nationwide epidemiologic study of norovirus-related hospitalization among Japanese older adults

**DOI:** 10.1186/s12879-019-4007-2

**Published:** 2019-05-09

**Authors:** Satoko Ohfuji, Kyoko Kondo, Kazuya Ito, Tetsuo Kase, Akiko Maeda, Wakaba Fukushima, Taisei Masuda, Munehide Kano

**Affiliations:** 1 Department of Public Health, Osaka City University Graduate School of Medicine, 1-4-3 Asahi-machi, Abeno-ku, Osaka, 545-8585 Japan; 2 Research Center for Infectious Disease Sciences, Osaka City University Graduate School of Medicine, 1-4-3 Asahi-machi, Abeno-ku, Osaka, 545-8585 Japan; 3 Administration Division, Osaka City University Hospital, 1-5-7 Asahi-machi, Abeno-ku, Osaka, 545-8586 Japan; 4 Global Vaccine Business Unit, Takeda Pharmaceutical Company Limited, 1-1 Nihonbashi-Honcho 2-chome, Chuo-ku, Tokyo, 103-8668 Japan

**Keywords:** Older adults, Epidemiology, Hospitalization, Mortality, Norovirus gastroenteritis

## Abstract

**Background:**

Older adults are vulnerable to hospitalization or death from norovirus infection, but the actual disease burden remains unknown. Therefore, we conducted a nationwide survey to estimate the number of inpatients with norovirus gastroenteritis and associated deaths among Japanese older adults.

**Methods:**

We performed a nationwide two-step query targeting 4184 hospital departments selected from 17,575 departments using stratified random sampling according to the number of beds. We asked each department to complete a mail-back questionnaire on the annual numbers of inpatients with infectious gastroenteritis and associated deaths between administrative years 2012 and 2014, and the implementation status of norovirus infection testing. In a second query, we investigated the annual number of inpatients with norovirus gastroenteritis and associated deaths in departments that had reported infectious gastroenteritis inpatients in the first query. Clinical information was collected for inpatients with norovirus gastroenteritis in administrative year 2014.

**Results:**

Norovirus testing for patients hospitalized for acute gastroenteritis was routinely conducted in 16% of the responding departments. Although half the departments responded that some acute gastroenteritis inpatients received such testing but others did not. In this situation, numbers of inpatients with norovirus gastroenteritis in Japan were estimated as 31,800 (95% CI: 25,700-37,900) in administrative year 2012, 21,600 (95% CI: 17,700–25,500) in administrative year 2013, and 15,700 (95% CI: 12,900–18,500) in administrative year 2014. The estimated number of associated deaths was approximately 600 in each administrative year. Factors associated with death included higher age, living in long-term care facilities, underlying illnesses such as chronic respiratory diseases, and complications such as aspiration pneumonia.

**Conclusions:**

The actual number of norovirus inpatient would be higher than the estimated here due to the low rate of routinely implemented norovirus testing. Considering Japan’s rapidly aging society and the disease burden of norovirus infection among Japanese older adults, it is important to protect this high-risk population from norovirus infection.

**Electronic supplementary material:**

The online version of this article (10.1186/s12879-019-4007-2) contains supplementary material, which is available to authorized users.

## Background

Noroviruses typically circulate from late fall through winter and causes gastrointestinal symptoms such as vomiting, diarrhea, and abdominal pain. The symptoms are generally mild and resolve within a few days. In older adults, however, norovirus infection can be serious and in some cases can lead to death from pulmonary aspiration of vomit. A 2016 review found hospitalization for norovirus and associated medical expenses and mortality rates are high in older adults [1]. As Japan is rapidly becoming a “super-aging” society, measures to maintain the health of older people are crucial [2]. However, there are currently no therapeutic medications for norovirus gastroenteritis, and symptomatic therapy is the main treatment. For these reasons, vaccines to prevent norovirus infection are under development [3].

In order to introduce vaccines, accurate information about the disease burden of norovirus gastroenteritis is needed. In Japan, the incidence of infectious gastroenteritis can be estimated from disease surveillance data obtained as part of the National Epidemiological Surveillance of Infectious Diseases [3]. However, the surveillance of infectious gastroenteritis is based on sentinel surveillance reports from pediatric hospital departments, and so it is not possible to estimate the potential number of older adults affected by norovirus. Furthermore, infectious gastroenteritis includes not only norovirus gastroenteritis, but also infections by other viruses such as rotavirus, adenovirus, and sapovirus, as well as bacterial infections [4, 5]. Consequently, the number of patients infected with each pathogen is unknown. Moreover, many cases of infectious gastroenteritis resolve within a few days so it is unusual for a diagnostic examination to be conducted in typical outpatient settings.

Therefore we conducted a nationwide epidemiologic study in Japan to assess the number of Japanese older adults hospitalized for infectious gastroenteritis and norovirus gastroenteritis and to clarify the clinical and epidemiologic features of these patients.

## Methods

The study was conducted in accordance with existing procedures proposed by the Research Committee on Epidemiology of Intractable Diseases in Japan [6]. The general method has been described previously [7–9]. The study consisted of two queries: the first intended to estimate the number of inpatients with infectious gastroenteritis among older adults, and the second to estimate the number of inpatients with norovirus gastroenteritis and to clarify their clinical and epidemiologic features.

### First query

A number of hospital departments were selected using stratified random sampling from a total of 17,575 departments of internal medicine, digestive diseases, gastroenterology, respiratory diseases, and cardiovascular diseases nationwide. These are the departments in which older people with aggravated infectious gastroenteritis predominantly receive treatment in Japan. The sampling was stratified by the number of hospital beds; sampling proportions were as follows: general hospitals with 99 beds or fewer, 5%; 100–199 beds, 10%; 200–299 beds, 20%; 300–399 beds, 40%; 400–499 beds, 80%; 500 beds or more, 100%; and university hospitals, 100%. A final total of 4184 departments were selected (Additional file 1: Table S1).

In January 2016, we asked these departments to complete a mail-back questionnaire, which was designed to ascertain the presence or absence of hospitalized patients with infectious gastroenteritis among adults aged ≥60 years between administrative years 2012 and 2014 (i.e., between April 1, 2012, and March 31, 2015). The information of the hospitalized patients included both community-acquired infection and nosocomial infection. We obtained data by administrative year, as hospital reporting is typically done in this manner in Japan. If present, the number of inpatients and deaths in each administrative year were additionally solicited. We also collected information on whether norovirus tests were routinely conducted at the department (routinely conducted, often conducted, or not conducted). A reminder was mailed to non-respondents in April 2016.

### Second query

In September 2016, we sent a second query to departments that had responded “yes” to the presence of patients hospitalized for infectious gastroenteritis in the first query. The second query was to collect data on the numbers of inpatients and deaths of those diagnosed with norovirus gastroenteritis among adults aged ≥60 years between administrative years 2012 and 2014. Determination of norovirus gastroenteritis depended on the physician’s diagnosis of each hospital. Clinical diagnosis without viral examination such as possible epidemiological link was allowed for counting number of norovirus gastroenteritis. To take into account the nosocomial infected cases, cases of norovirus gastroenteritis diagnosed not only at admission but also during the hospital stay were included. For patients hospitalized in administrative year 2014, the following clinical information was also collected: birth month and birth year, sex, residence at the onset of gastroenteritis (i.e., home, long-term care facility, or hospital), underlying diseases (hypertension, heart disease, diabetes, stroke, malignant tumor, renal disease, chronic respiratory disease, liver disease, hematological disorders, or collagen diseases), date of admission, date of discharge, possible cause of infection based on the physician’s medical examination including interview to the patients (contaminated food, contact with infected person(s), disease epidemic in the area), results of tests for norovirus, clinical symptoms (presence, frequency, and duration of diarrhea and vomiting; presence of fever), date of symptom onset and duration, complicated diseases (such as aspiration pneumonia), laboratory data at the time of admission, treatment (e.g., intravenous drip, antibiotics, and intensive care unit therapy), clinical outcome (recovered, moved to another hospital, self-discharged, or deceased), and cause of death if deceased. We assumed that the clinical information of norovirus gastroenteritis did not change significantly between administrative years 2012 and 2014, and due to research budget constraints we did not obtain the clinical information for patients hospitalized in administrative years 2012 and 2013, but choose to investigate the latest information available, i.e. in administrative year 2014. We confirmed our assumption that the epidemic trend of norovirus infection in the three administrative years was stable in Japan using national surveillance data [10]. We mailed a reminder to non-respondents in November 2016. Additionally, we asked the departments that had responded to confirm or revise those parts of the previously returned questionnaire that had missing or conflicting information. If there were missing data even after confirmation or revision, the data were considered incomplete.

### Statistical analysis

Accounting for sampling and response proportions in the first query, we estimated the total numbers of inpatients and deaths due to infectious gastroenteritis among adults aged ≥60 years between administrative years 2012 and 2014 according to the following formula: estimated total number of inpatients = reported number of inpatients / (sampling proportion × response proportion). Additionally, 95% confidence intervals (CIs) were calculated with an assumption of multinomial hypergeometric distribution [6–9].

We also estimated the total numbers of inpatients and deaths due to norovirus gastroenteritis among adults aged ≥60 years between administrative years 2012 and 2014 using data from the second query. In this calculation, we used the following formula: estimated total number of inpatients with norovirus gastroenteritis = estimated total number of inpatients with infectious gastroenteritis × proportion of reported number of inpatients with norovirus gastroenteritis among the reported number of inpatients with infectious gastroenteritis. This latter proportion was based on information from departments that responded to the second query. Total estimated numbers and 95% CIs were rounded to three significant digits, except for the hundreds, which were rounded to two significant digits.

Hospitalization and mortality rates in each administrative year were calculated using the number of Japanese people aged ≥60 years at October 1 in each administrative year (i.e., 41,038,000 in administrative year 2012, 41,561,000 in administrative year 2013, and 41,980,000 in administrative year 2014) [11–13].

The clinical characteristics of inpatients with norovirus gastroenteritis were also examined. Age at admission was calculated using information on birth month and birth year and date of admission. If the admission date had not been recorded, it was regarded as October 1 for the calculation. Disease severity was assessed using modified Vesikari scores [14]. In order to assess the disease severity using laboratory data such data were categorized into two or three levels according to standard values for the Japanese population [15]. To examine factors associated with death, a logistic regression model was used to obtain odds ratios (ORs) and 95% CIs. Since age and sex are the important predictors for death, these variables were included as co-factors in a logistic regression model.

All tests were two-sided. All analyses were performed using SAS version 9.3 software (SAS Institute, Cary, NC, USA).

## Results

In the first query, 1325 out of 4184 departments responded (response proportion: 31.7%). Among these, 561 departments reported the presence of inpatients with infectious gastroenteritis; numbers of reported inpatients were 9857 in administrative year 2012, 8361 in administrative year 2013, and 8410 in administrative year 2014 (Additional file 1: Table S1). The response rates from gastroenterology departments in large hospitals were low (e.g. 20% in five University hospitals, 0% in four ≥500 beds hospitals). Since there were only nine gastroenterology departments, the low response rate did not have a major impact on the present results (Additional file 1: Table S1). Based on the results of the first query, the numbers of inpatients with infectious gastroenteritis among Japanese adults aged ≥60 years were estimated to be 118,000 (95% CI: 95,700–141,000) in administrative year 2012, 95,100 (95% CI: 77,700–112,000) in administrative year 2013, and 96,900 (95% CI: 79,500–114,000) in administrative year 2014. Infectious gastroenteritis was estimated to have caused 2060 (95% CI: 1370–2750), 1940 (95% CI: 1230–2640), and 1970 (95% CI: 1280–2650) deaths among Japanese adults aged ≥60 years in administrative years 2012, 2013, and 2014, respectively (Table 1).

**Table 1:** Estimated number of hospitalized patients and deaths due to infectious gastroenteritis among adults aged ≥60 years in Japan

Stratum (No. of hospital beds)	Administrative year 2012		Administrative year 2013		Administrative year 2014	
	Hospitalized patients	Deaths	Hospitalized patients	Deaths	Hospitalized patients	Deaths
University hospital	1366	16	1251	30	1119	14
≥500	9502	469	8152	461	7684	415
400–499	9152	181	7797	180	8310	182
300–399	20,879	470	17,919	448	19,072	583
200–299	18,365	133	15,640	119	15,200	191
100–199	39,120	661	28,289	697	27,879	512
< 99	20,056	130	16,012	0	17,632	74
Total estimated number	118,000	2060	95,100	1940	96,900	1970
(95% confidence interval)	(95,700–141,000)	(1370–2750)	(77,700–112,000)	(1230–2640)	(79,500–114,000)	(1280–2650)

In the second query, 271 out of 561 departments responded (response proportion: 48.0%), and 126 departments reported the presence of inpatients with norovirus gastroenteritis. Among these departments, the proportions of reported number of inpatients with norovirus gastroenteritis among the reported number of inpatients with infectious gastroenteritis were 26.9% in administrative year 2012, 22.7% in administrative year 2013, and 16.2% in administrative year 2014. Thus, the estimated numbers of inpatients with norovirus gastroenteritis among Japanese adults aged ≥60 years were calculated as 31,800 (95% CI: 25,700–37,900) in administrative year 2012, 21,600 (95% CI: 17,700–25,500) in administrative year 2013, and 15,700 (95% CI: 12,900–18,500) in administrative year 2014 (Table 2). The hospitalization rates (per 10,000 persons) were 7.75, 5.20, and 3.74, respectively. Among the reported number of deaths from infectious gastroenteritis 31.3% in administrative year 2012, 30.0% in administrative year 2013, and 29.6% in administrative year 2014 were due to norovirus, and norovirus gastroenteritis was estimated to have caused a total of 650 (95% CI: 430–860), 580 (95% CI: 370–790), and 580 (95% CI: 380–790) deaths in administrative years 2012, 2013, and 2014, respectively (Table 2). The respective mortality rates (per 100,000 persons) were 1.58, 1.40, and 1.38.

**Table 2:** Estimated number of hospitalized patients and deaths due to norovirus gastroenteritis among adults aged ≥60 years in Japan

Stratum (No. of hospital beds)	Administrative year 2012		Administrative year 2013		Administrative year 2014	
	Hospitalized patients	Deaths	Hospitalized patients	Deaths	Hospitalized patients	Deaths
University hospital	367	5	284	9	181	4
≥500	2552	147	1853	138	1246	123
400–499	2458	57	1772	54	1347	54
300–399	5608	147	4072	135	3092	172
200–299	4932	42	3554	36	2464	56
100–199	10,507	207	6429	209	4519	151
< 99	5387	41	3639	0	2858	22
Total estimated number	31,800	650	21,600	580	15,700	580
(95% confidence interval)	(25,700–37,900)	(430–860)	(17,700–25,500)	(370–790)	(12,900–18,500)	(380–790)

**Fig. 1:**
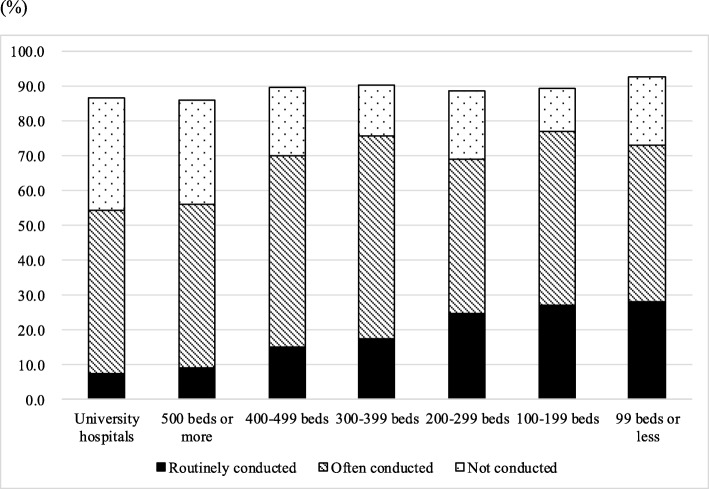
Implementation status of norovirus infection testing at university hospitals and general hospitals according to number of hospital beds. According to answers of “unknown data”, each total has not reached 100%

Figure 1 shows the implementation status of norovirus infection testing in Japanese hospitals. Approximately 15.8% of departments replied that norovirus testing (mainly rapid antigen testing) was “routinely conducted” for patients hospitalized for acute gastroenteritis; 49.7% of departments responded that such testing was “often conducted”, which means that some acute gastroenteritis inpatients received norovirus testing but other acute gastroenteritis inpatients did not; 22.9% of departments did not conduct norovirus tests for patients hospitalized for gastroenteritis. In particular, university hospitals or general hospitals with larger numbers of beds tended to respond “not conducted.”

Table 3 summarizes the clinical characteristics of inpatients with norovirus gastroenteritis in administrative year 2014. Male patients accounted for 42% of inpatients. Approximately 55% of patients were aged ≥80 years. Most patients (90%) had underlying illnesses. Regarding the possible cause of infection, only 9% were suspected to be caused by contaminated food, and 21% were presumably caused by contact with an infected person considered to be possible epidemiological link. However, the cause was not known for about half of the patients. Only 61% of inpatients received norovirus testing. Among those, half had positive results. Most patients had diarrhea and half had symptoms of vomiting or fever. Approximately 6% of cases were complicated by aspiration pneumonia. The median duration of hospital admission was 11 days; although 93% of patients recovered, 4% died. The main cause of death was pneumonia (*n* = 7). Other causes were as follows: heart failure (*n* = 4), malignant tumor (n = 4), and ileus, digestive tract hemorrhage, respiratory failure, multi-organ failure, and perforation of the digestive tract (*n* = 1 each).

**Table 3:** Characteristics of patients hospitalized for norovirus gastroenteritis (*N* = 470)

Characteristics		n (%) or median (range)^a^	Characteristics		n (%) or median (range)^a^
Sex	Male	194 (42)	Laboratory data at the time of admission		
Age (years)	60–69	66 (14)	White blood cell (/μL)	Decreased	17 (4)
	70–79	145 (31)		Normal	231 (51)
	≥80	259 (55)		Increased	202 (45)
Residence at symptom onset			Hemoglobin (g/dL)	Decreased	190 (42)
	Home	287 (62)		Normal	249 (55)
	Long-term care facility	62 (13)		Increased	17 (4)
	Hospital	115 (25)	Platelet count (× 10^4^/μL)	Decreased	47 (10)
Underlying illnesses		424 (90)		Normal	386 (84)
	Hypertension	213 (45)		Increased	28 (6)
	Heart disease	121 (26)	C-reactive protein (mg/dL)	Increased	349 (77)
	Diabetes	106 (23)	Blood sugar (mg/dL)	Decreased	18 (5)
	Stroke	121 (26)		Normal	102 (26)
	Malignant tumor	66 (14)		Increased	268 (69)
	Renal disease	38 (8)	Albumin (g/dL)	Decreased	230 (59)
	Chronic respiratory disease	32 (7)	Aspartate aminotransferase (IU/L)	Increased	205 (45)
	Liver disease	24 (5)	Creatinine (mg/dL)	Increased	200 (44)
	Hematological disorders	13 (3)	Sodium (mEq/L)	Decreased	81 (18)
	Collagen diseases	7 (1)		Normal	375 (82)
Possible causes of infection				Increased	4 (1)
	Contaminated food	43 (9)	Potassium (mEq/L)	Decreased	106 (23)
	Contact with infected person(s)	95 (21)		Normal	329 (72)
	Disease epidemic in the area	99 (21)		Increased	24 (5)
Tested for norovirus		287 (61)	Chloride (mEq/L)	Decreased	54 (12)
Test result	Positive	153 (53)		Normal	376 (83)
Clinical symptoms at the time of admission				Increased	23 (5)
Diarrhea	Present	377 (80)	Treatment		
	Duration (days)	4 (1–36)^a^	Intravenous drip		386 (82)
	Frequency per day	3.5 (1–33)^a^	Antibiotics		201 (43)
Vomiting	Present	232 (49)	Oxygen supplementation		73 (16)
	Duration (days)	2 (1–14)^a^	Intensive care unit therapy		10 (2)
	Frequency per day	1.5 (1–25)^a^	Use of respirator		7 (1)
Fever	Present	262 (56)	Duration of hospital admission (days)		11 (1–2359)^a^
	Maximum fever (°C)	37.85 (37.0–40.3)^a^	Clinical outcome	Recovered	438 (93)
Complications	Aspiration pneumonia	28 (6)		Moved to hospital	10 (2)
	Others	35 (8)		Deceased	21 (4)
Modified Vesikari scale		8 (0–16)^a^		Self-discharged	1 (0.2)

^a^median (range)

Higher mortality in 470 clinical diagnosed cases was observed among males, older patients, those living in long-term care facilities, those with particular underlying illnesses such as chronic respiratory diseases, and those with complications such as aspiration pneumonia (Table 4). Female patients had a significantly decreased adjusted OR (aOR) for death compared with male patients (aOR = 0.41, 95% CI: 0.16–1.00). Moreover, those living in long-term care facilities (aOR = 3.39, 95% CI: 1.15–9.55), those with chronic respiratory diseases (aOR = 3.90, 95% CI: 1.01–12.5), and those with aspiration pneumonia (aOR = 8.97, 95% CI: 3.06–24.7) had significantly increased aORs for death.

**Table 4:** Association between selected background characteristics and death from norovirus gastroenteritis^a^

Characteristics		Mortality	Univariate analysis	Age/sex-adjusted analysis
		n/N (%)	OR (95% CI)	aOR (95% CI)
Sex	Male	12/194 (6)	1.00	1.00
	Female	9/273 (3)	0.52 (0.21–1.25)	0.41 (0.16–1.00)
Age (years)	60–69	2/66 (3)	1.00	1.00
	70–79	3/145 (2)	0.68 (0.11–5.22)	0.69 (0.11–5.35)
	≥80	16/259 (6)	2.11 (0.58–13.5)	2.63 (0.71–17.1)
			(Trend *P* = 0.11)	(Trend *P* = 0.05)
Residence at symptom onset	Home	11/287 (4)	1.00	1.00
	Long-term care facility	7/62 (11)	3.19 (1.13–8.48)	3.39 (1.15–9.55)
	Hospital	3/115 (3)	0.67 (0.15–2.20)	0.67 (0.15–2.22)
Underlying illnesses	Absent	1/45 (2)	1.00	1.00
	Present	20/424 (5)	2.18 (0.44–39.5)	2.16 (0.43–39.4)
Chronic respiratory disease	Absent	17/438 (4)	1.00	1.00
	Present	4/32 (13)	3.54 (0.97–10.4)	3.90 (1.01–12.5)
Collagen diseases	Absent	20/463 (4)	1.00	1.00
	Present	1/7 (14)	3.69 (0.19–23.1)	4.11 (0.21–28.1)
Hematological disorders	Absent	19/457 (4)	1.00	1.00
	Present	2/13 (15)	4.19 (0.62–17.1)	4.40 (0.64–18.9)
Complications				
Aspiration pneumonia	Absent	14/439 (3)	1.00	1.00
	Present	7/28 (25)	10.1 (3.53–27.2)	8.97 (3.06–24.7)
Others	Absent	15/427 (4)	1.00	1.00
	Present	6/35 (17)	5.68 (1.91–15.2)	5.42 (1.79–14.8)

*Abbreviations*: *OR* odds ratio, *aOR* adjusted odds ratio, *CI* confidence interval

^a^Logistic regression model

## Discussion

The results of our study suggest that approximately 100,000 persons aged ≥60 years are hospitalized for infectious gastroenteritis annually in Japan, of which approximately one-quarter are hospitalized for norovirus gastroenteritis. The number of inpatients with norovirus gastroenteritis was approximately 15,000 in administrative year 2014 (when the disease occurred at a low rate) and approximately 30,000 in administrative year 2012 (when it became epidemic, with approximately 600 annual deaths). The annual hospitalization rate (per 10,000 population) for norovirus gastroenteritis was 3.74–7.75 and the annual mortality rate (per 100,000 population) was 1.38–1.58 among persons aged ≥60 years. However, since the proportion of departments which routinely conducted norovirus testing for acute gastroenteritis inpatients was lower than expected, the actual number of norovirus inpatients would be higher than the estimated number here.

The present study showed a large number of inpatients in administrative year 2012 and relatively few inpatients in administrative year 2014, and this trend was consistent with the National Surveillance Data of infectious gastroenteritis patients reported by sentinels [4]. Additionally, the proportion of norovirus inpatients among infectious gastroenteritis inpatients in the present study (16–27%) was similar to that reported in a meta-analysis of 175 research papers (17–20%) [16]. However, the proportion was somewhat lower than the proportions reported by other studies in Japan (34–39%) [17–19]. As norovirus gastroenteritis is an infectious disease, its epidemic status varies between geographical regions. It is possible therefore that the differences in the proportion of norovirus patients between the present study and other studies in Japan merely indicate differences in epidemic status among geographical regions.

In Japan, norovirus testing is not routinely conducted by all hospitals so it is possible that the results of our study were influenced by the status of implementation of testing at the hospitals that were surveyed. In fact, institutions not testing for norovirus accounted for one-quarter of the hospitals that were studied. In the present study, it is possible that such institutions reported having no inpatients with norovirus gastroenteritis. Therefore, the present data may underestimate the annual number of inpatients with norovirus gastroenteritis and also the proportion of nosocomial infection. In addition, university hospitals or general hospitals with larger numbers of beds tended to report that they had not conducted norovirus testing. We assumed that these hospitals were more likely to contain patients with infectious gastroenteritis and assigned a higher sampling proportion to these hospitals in the study protocol. However, the unexpectedly lower implementation status of norovirus testing might have affected the study results. One study that estimated the burden of norovirus gastroenteritis in Japan using a modelling approach accounting for the absence of routine diagnostic testing showed higher incidence rates in the elderly than our study [20]. However, the database used in that study had relatively few older adults and therefore may not have been representative of the Japanese population. Compared with the previous study, our findings seem to suggest a lower norovirus disease burden in older adults according to laboratory-confirmed cases. This represents a possible limitation of our study in that many hospitals did not conduct norovirus testing. Another limitation was a lower response proportion to the first query (31.7%), which may have introduced bias. According to a nationwide epidemiologic investigation manual issued by the Japanese Ministry of Health, Labour and Welfare [5], a response proportion of approximately 60% can produce a reliable estimation of number of patients; however, we did not obtain this response proportion. If non-response were associated with nosocomial norovirus infection due to the hesitancy to disclose negative clinical practice in hospitals, the present results might be biased. In general, however, medical institutes that are highly conscious of measures against infectious diseases, such as having their own manual for infectious diseases, are likely to have responded in our study, and their responses seemed to be highly reliable. On the other hand, in the non-response departments, norovirus gastroenteritis may not be sufficiently understood. In the present study protocol, assuming that non-response departments had a similar proportion of inpatients with norovirus gastroenteritis as those departments which responded, we estimated the number of inpatients or deaths with norovirus gastroenteritis in Japan. However, we cannot deny the possibility that precision of the present results may be low, although we believe that the present results may be validated.

The annual hospitalization and mortality rates for norovirus gastroenteritis in the present study are similar to those in other countries. A systematic review of 39 studies in adults aged > 65 years worldwide reported the hospitalization rate for norovirus gastroenteritis as 1–19 per 10,000 persons and the mortality rate as 0.4–3.2 per 100,000 persons [21]. Our results fall within these ranges. The systematic review also reported the annual rate of outpatient visits for norovirus gastroenteritis as 18–54 per 10,000 persons and the annual incidence rate as 29–125 per 10,000 persons. In this study, we did not obtain information on outpatient visits and incidence; thus, we cannot compare these rates. If the results of the systematic review are applied to Japan, the number of outpatient visits for norovirus gastroenteritis among the population aged ≥60 years (42.75 million, October 2016) would be estimated at 76,950–230,850, and the annual number of incident cases would be estimated at 123,975–534,375, suggesting that norovirus gastroenteritis has a substantial influence on the health of Japanese older adults.

We found that the possible cause of norovirus gastroenteritis was consumption of contaminated food in 9% of inpatients and person-to-person transmission in 21%. Compared with an outbreak investigation conducted in Spain, the present study found a higher proportion of cases of unknown cause; in the Spanish outbreak investigation, 42% of cases were probably caused by consumption of contaminated food and 52% were likely caused by person-to-person transmission [22]. The present results highlight the difficulties in determining the cause of norovirus gastroenteritis infection occurring in the community.

Regarding clinical symptoms, diarrhea was reported in 80% of cases, vomiting in 49%, and fever in 56%. These proportions are lower than those reported in pediatric studies. In young children in an Israeli study diarrhea was reported in 81%, vomiting in 86%, and fever in 64% of patients [23]. Disease severity was lower in the present study than in a study of young children conducted in Taiwan, which reported a median Vesikari value of 12.5 [24]. However, intravenous drip was more often administered to inpatients in the present study compared with children in the Israeli study (82% vs. 68%) [23]. The median duration of hospital admission was longer in the present study compared with children in the Taiwanese study (11 days vs. 3 days) [24]. Differences in treatment may simply reflect differences in medical care between study regions. Nonetheless, the present results also suggest that, compared with children, older adults tend to experience more dehydration and require more time to recover. Such information may be important when the disease burden of norovirus gastroenteritis is considered from a medical economics perspective.

In our study, 4% of hospitalized patients died. Factors associated with death include greater age, living in long-term care facilities, underlying illnesses (particularly chronic respiratory diseases), and complications such as aspiration pneumonia. In a study of 1877 cases in an outbreak of norovirus gastroenteritis in England and Wales between 1992 and 2000, all deaths from norovirus gastroenteritis occurred in patients who were hospitalized or institutionalized [25]. A systematic review of reports from 1988 to 2011 found more deaths among older adults, those living in long-term care facilities, and those in an immunosuppressed state, and the most common cause of death was aspiration pneumonia (32%) [26]. Older adults with underlying illnesses (particularly chronic respiratory diseases) are considered to be highly susceptible to infectious diseases, which may lead to a high risk of death when norovirus gastroenteritis develops as a complication. Taken together, the factors associated with death in the present study are consistent with these previous reports. Therefore, we hope that these results will provide useful information for the future introduction of vaccine measures.

## Conclusions

In conclusion, although the actual number of norovirus inpatients is probably higher than the estimates here due to the low rate of routinely implemented norovirus testing, the annual number of patients hospitalized for norovirus gastroenteritis was estimated to range from 15,700 (administrative year 2014) to 31,800 (administrative year 2012), and the annual number of deaths was estimated to be between 580 (administrative years 2013, 2014) and 650 (administrative year 2012). The annual hospitalization rate was 3.74 (administrative year 2014) to 7.75 (administrative year 2012) per 10,000 persons, and the annual mortality rate was 1.38 (administrative year 2014) to 1.58 (administrative year 2012) per 100,000 persons. The factors associated with death among inpatients with norovirus gastroenteritis included higher age, living in long-term care facilities, underlying illnesses (particularly chronic respiratory diseases), and complications such as aspiration pneumonia. Considering Japan’s rapidly aging society and the disease burden of norovirus infection among Japanese older adults, it is important to protect this high-risk population from norovirus infection.

## Additional file

Additional file 1: **Table S1.** The numbers of reported inpatients and reported deaths due to infectious gastroenteritis among Japanese older adults: results of the first query. (DOCX 24 kb)

## Abbreviations

aOR: Adjusted odds ratio
CI: Confidence interval
OR: Odds ratio
