# Supplementary material for: Nationwide epidemiologic study of norovirus-related hospitalization among Japanese older adults
Source: BMC Infect Dis. 2019 May 9;19:400. doi: 10.1186/s12879-019-4007-2 (PMC6506929; doi:10.1186/s12879-019-4007-2)
Supplement: Supplementary file 1 — Table S1. The numbers of reported inpatients and reported deaths due to infectious gastroenteritis among Japanese older adults: results of the first query. (DOCX 24 kb) [file 12879_2019_4007_MOESM1_ESM.docx]

**Table S1. The numbers of reported inpatients and reported deaths due to infectious gastroenteritis among Japanese older adults: results of the first query.**

| Department | Stratum (No. of hospital beds) | No. of departments | No. of selected departments | Sampling proportion | No. of responded departments | Response proportion | Fiscal 2012 | | Fiscal 2013 | | Fiscal 2014 | |
| --- | --- | --- | --- | --- | --- | --- | --- | --- | --- | --- | --- | --- |
|  |  |  |  |  |  |  | No. of reported inpatients | No. of reported deaths | No. of reported inpatients | No. of reported deaths | No. of reported inpatients | No. of reported deaths |
| Internal medicine | University hospital | 137 | 137 | (100.0%) | 46 | (33.6%) | 290 | 4 | 222 | 5 | 217 | 2 |
|  | ≥500 beds | 312 | 312 | (100.0%) | 75 | (24.0%) | 1,296 | 69 | 1,203 | 78 | 1,033 | 63 |
|  | 400-499 | 340 | 272 | (80.0%) | 79 | (29.0%) | 1,016 | 14 | 808 | 15 | 942 | 17 |
|  | 300-399 | 628 | 250 | (39.8%) | 65 | (26.0%) | 1,087 | 39 | 896 | 36 | 981 | 47 |
|  | 200-299 | 943 | 188 | (19.9%) | 50 | (26.6%) | 367 | 3 | 264 | 0 | 295 | 3 |
|  | 100-199 | 2,442 | 244 | (10.0%) | 75 | (30.7%) | 320 | 7 | 346 | 17 | 269 | 8 |
|  | < 99 beds | 2,623 | 132 | (5.0%) | 47 | (35.6%) | 65 | 1 | 62 | 0 | 76 | 0 |
|  | Subtotal | 7,425 | 1,535 | (20.7%) | 437 | (28.5%) | 4,441 | 137 | 3,801 | 151 | 3,813 | 140 |
| Digestive Diseases | University hospital | 126 | 126 | (100.0%) | 63 | (50.0%) | 150 | 0 | 182 | 2 | 161 | 0 |
|  | ≥500 beds | 215 | 215 | (100.0%) | 59 | (27.4%) | 1,031 | 43 | 791 | 36 | 863 | 39 |
|  | 400-499 | 201 | 161 | (80.1%) | 46 | (28.6%) | 748 | 23 | 692 | 21 | 677 | 15 |
|  | 300-399 | 306 | 122 | (39.9%) | 34 | (27.9%) | 875 | 4 | 854 | 8 | 862 | 9 |
|  | 200-299 | 353 | 71 | (20.1%) | 25 | (35.2%) | 228 | 3 | 248 | 6 | 239 | 6 |
|  | 100-199 | 969 | 97 | (10.0%) | 27 | (27.8%) | 442 | 11 | 255 | 4 | 319 | 7 |
|  | < 99 beds | 940 | 47 | (5.0%) | 14 | (29.8%) | 18 | 0 | 16 | 0 | 21 | 0 |
|  | Subtotal | 3,110 | 839 | (27.0%) | 268 | (31.9%) | 3,492 | 84 | 3,038 | 77 | 3,142 | 76 |
| Gastroenterology | University hospital | 5 | 5 | (100.0%) | 1 | (20.0%) | 0 | 0 | 0 | 0 | 0 | 0 |
|  | ≥500 beds | 4 | 4 | (100.0%) | 0 | (0.0%) | 0 | 0 | 0 | 0 | 0 | 0 |
|  | 400-499 | 8 | 7 | (87.5%) | 2 | (28.6%) | 0 | 0 | 0 | 0 | 0 | 0 |
|  | 300-399 | 30 | 12 | (40.0%) | 3 | (25.0%) | 85 | 0 | 5 | 0 | 10 | 0 |
|  | 200-299 | 52 | 11 | (21.2%) | 3 | (27.3%) | 17 | 0 | 4 | 0 | 5 | 0 |
|  | 100-199 | 312 | 32 | (10.3%) | 9 | (28.1%) | 69 | 0 | 27 | 0 | 30 | 0 |
|  | < 99 beds | 592 | 30 | (5.1%) | 8 | (26.7%) | 71 | 1 | 81 | 0 | 83 | 1 |
|  | Subtotal | 1,003 | 101 | (10.1%) | 26 | (25.7%) | 242 | 1 | 117 | 0 | 128 | 1 |
| Respiratory Diseases | University hospital | 120 | 120 | (100.0%) | 63 | (52.5%) | 64 | 2 | 67 | 6 | 44 | 4 |
|  | ≥500 beds | 211 | 211 | (100.0%) | 89 | (42.2%) | 75 | 4 | 64 | 0 | 49 | 0 |
|  | 400-499 | 194 | 155 | (79.9%) | 53 | (34.2%) | 249 | 2 | 210 | 3 | 220 | 7 |
|  | 300-399 | 273 | 108 | (39.6%) | 35 | (32.4%) | 15 | 0 | 28 | 0 | 27 | 0 |
|  | 200-299 | 295 | 58 | (19.7%) | 15 | (25.9%) | 91 | 1 | 104 | 1 | 71 | 1 |
|  | 100-199 | 719 | 71 | (9.9%) | 10 | (14.1%) | 74 | 0 | 30 | 0 | 29 | 0 |
|  | < 99 beds | 620 | 31 | (5.0%) | 11 | (35.5%) | 60 | 0 | 36 | 0 | 18 | 0 |
|  | Subtotal | 2,432 | 754 | (31.0%) | 276 | (36.6%) | 628 | 9 | 539 | 10 | 458 | 12 |
| Cardiovascular Diseases | University hospital | 127 | 127 | (100.0%) | 57 | (44.9%) | 36 | 0 | 44 | 0 | 30 | 0 |
|  | ≥500 beds | 244 | 244 | (100.0%) | 93 | (38.1%) | 67 | 6 | 43 | 2 | 48 | 4 |
|  | 400-499 | 227 | 182 | (80.2%) | 53 | (29.1%) | 140 | 3 | 123 | 3 | 115 | 4 |
|  | 300-399 | 379 | 150 | (39.6%) | 40 | (26.7%) | 162 | 6 | 138 | 3 | 161 | 5 |
|  | 200-299 | 429 | 85 | (19.8%) | 29 | (34.1%) | 415 | 1 | 341 | 1 | 323 | 2 |
|  | 100-199 | 1,156 | 114 | (9.9%) | 30 | (26.3%) | 133 | 1 | 124 | 0 | 118 | 0 |
|  | < 99 beds | 1,043 | 53 | (5.1%) | 16 | (30.2%) | 101 | 0 | 53 | 0 | 74 | 0 |
|  | Subtotal | 3,605 | 955 | (26.5%) | 318 | (33.3%) | 1,054 | 17 | 866 | 9 | 869 | 15 |
|  | Total | 17,575 | 4,184 | (23.8%) | 1,325 | (31.7%) | 9,857 | 248 | 8,361 | 247 | 8,410 | 244 |
